# Bridging maternal effects and epitranscriptomics: A novel perspective in developmental biology

**DOI:** 10.1002/dvdy.70111

**Published:** 2025-12-30

**Authors:** Ehsan Pashay Ahi

**Affiliations:** ^1^ Organismal and Evolutionary Biology Research Programme, Faculty of Biological and Environmental Sciences University of Helsinki Helsinki Finland

**Keywords:** developmental programming, epitranscriptomics, maternal effects, non‐genetic inheritance, RNA modifications

## Abstract

Maternal effects, encompassing both genetic (maternally expressed gene products) and non‐genetic (maternal state) influences, are powerful determinants of offspring phenotype, yet their RNA‐level mechanisms remain incompletely resolved. In parallel, epitranscriptomics, an emerging field centered on chemical modifications to RNA, has revealed new layers of gene regulation with implications for cell fate, plasticity, and response to environmental cues. In this perspective article, a conceptual link is proposed between maternal effects and epitranscriptomic mechanisms, focusing on how maternal environments may shape offspring phenotypes through RNA modifications. Evidence is examined from diverse systems, including maternal deposition of modified RNAs, environmental modulation of RNA‐modifying enzymes, and early developmental windows sensitive to maternal inputs. A clear distinction is drawn between placenta‐mediated pathways that reprogram trophoblast/placental epitranscriptomics and direct fetal‐tissue routes that act within developing organs. Although causal demonstrations are still emerging, convergent observations indicate that maternal environments can tune the offspring epitranscriptome with lasting phenotypic consequences. To articulate this emerging connection, the concept of “*maternal RNA imprinting*” is proposed, the idea that offspring development is shaped by maternal cues via targeted RNA modifications. This article aims not only to synthesize emerging insights across fields but also to stimulate interdisciplinary discussion and encourage investigation into the unexplored intersections of maternal biology and RNA regulation.

## 1 MATERNAL EFFECTS: BEYOND GENETIC INHERITANCE

Maternal effects have been increasingly recognized as important regulators of offspring phenotype, which can operate independently of the inherited DNA sequence.^1^, ^2^ Traditionally viewed through the lens of genetics, developmental outcomes were once largely attributed to allelic combinations passed from parents to progeny.^3^, ^4^ Classical forward genetic screens in Drosophila and, in zebrafish, four‐generation natural‐mating maternal‐effect screening strategies have identified numerous maternal‐effect mutations, including strict maternal‐effect alleles in which the mother's genotype alone determines embryonic phenotype via maternally deposited transcripts and proteins.^5^, ^6^, ^7^, ^8^, ^9^ More recently, CRISPR/Cas9‐based approaches such as maternal crispants, primordial germ cell transplantation‐based germline editing, oocyte‐specific Cas9 transgenes, and F0 null‐mutation strategies now enable rapid generation and analysis of maternal and maternal‐zygotic mutants in zebrafish, substantially expanding the accessible repertoire of vertebrate maternal‐effect genes.^10^, ^11^, ^12^, ^13^ However, it has become evident that the maternal environment exerts a substantial influence on offspring development through a variety of non‐genetic mechanisms.^14^, ^15^, ^16^, ^17^ These maternal effects encompass physiological, biochemical, and behavioral cues, transmitted during oogenesis, gestation, or early postnatal life.^3^, ^18^, ^19^

In animal systems, maternal effects have been well‐documented in contexts such as nutrition, stress exposure, metabolic state, and immune status.^20^, ^21^, ^22^, ^23^, ^24^, ^25^, ^26^, ^27^ These factors have been shown to influence offspring traits ranging from growth rate and metabolism to neurodevelopment and immune function. In many cases, such effects have persisted into adulthood and even across generations, suggesting involvement of mechanisms capable of inducing stable, long‐term changes in gene expression or cellular state.^18^, ^27^, ^28^, ^29^, ^30^, ^31^, ^32^ Importantly, these influences are often mediated through the maternal provisioning of molecular substrates during early development, including hormones, metabolites, proteins, and RNAs.^33^, ^34^, ^35^, ^36^, ^37^, ^38^ In addition to the classical maternal‐effect genetics in *Drosophila* and *zebrafish* defining extensive sets of maternally required loci,^5^, ^6^, ^7^, ^8^, ^9^ recent research has expanded the understanding of maternal effects to include the regulation of gene expression programs during embryogenesis.^35^, ^39^, ^40^, ^41^, ^42^, ^43^ Notably, maternal contributions have been implicated in modulating chromatin architecture and DNA methylation landscapes in the developing embryo.^44^, ^45^, ^46^, ^47^, ^48^ These epigenetic alterations offer a mechanistic explanation for how transient environmental exposures in the mother can produce sustained phenotypic outcomes in the offspring.^44^, ^49^, ^50^, ^51^ However, while considerable attention has been given to DNA‐level epigenetic marks, a growing body of evidence now points to RNA as another critical layer of regulatory control.

Maternal RNAs, transcripts deposited into the oocyte during oogenesis, serve as essential regulators of early embryonic development, particularly in organisms where zygotic genome activation (ZGA) is delayed (e.g., zebrafish and *Drosophila*; *ex ovo*), with RNA modifications on maternal mRNAs (notably m^6^A and m^5^C) directing their clearance and translation.^52^, ^53^, ^54^, ^55^, ^56^, ^57^, ^58^ These RNAs direct early cell divisions, patterning, and axis formation before the embryo begins transcribing its own genome.^59^, ^60^, ^61^ The stability, translation, and function of these transcripts can be influenced by post‐transcriptional modifications, for example, m^6^A and m^5^C, which are now understood to be dynamic and responsive to environmental conditions, as shown in zebrafish, *Drosophila*, Xenopus, and mammalian oocytes/embryos.^52^, ^55^, ^57^, ^58^, ^62^, ^63^, ^64^ As such, maternal effects may extend into the realm of post‐transcriptional regulation, potentially involving mechanisms such as RNA methylation or editing, with evidence from human preimplantation embryos (RNA editing) and from mouse oocytes/embryos (m^6^A), as well as cross‐species m^5^C maps of maternal mRNAs.^52^, ^65^, ^66^, ^67^ These findings raise the possibility that maternal effects may be exerted, in part, through modulation of the embryonic epitranscriptome.^68^, ^69^, ^70^ Though this concept remains underexplored, it introduces a compelling hypothesis: that maternal effects and epitranscriptomic regulation may intersect to form a unified system of non‐genetic developmental programming. By considering maternal effects as more than mere nutrient or hormonal signals, and by integrating recent discoveries from RNA biology, this article sets the stage for examining how RNA modifications may act as a mechanistic bridge linking maternal cues to offspring phenotype. This emerging perspective opens new avenues for research and invites a reevaluation of how maternal environments shape development at the molecular level.

## 2 EPITRANSCRIPTOMICS: ADDING REGULATORY DIMENSION TO RNA BIOLOGY

Epitranscriptomics, the study of post‐transcriptional chemical modifications on RNA molecules, has added an important new layer of regulatory complexity to gene expression. Unlike canonical epigenetics, which primarily targets DNA and histone proteins, epitranscriptomic modifications act directly on RNA, influencing its splicing, stability, localization, translation, and degradation.^71^, ^72^, ^73^, ^74^, ^75^, ^76^ More than 170 types of RNA modifications have been identified, with N6‐methyladenosine (m^6^A) the most prevalent on eukaryotic mRNA,^77^, ^78^ first reported on mRNA in the 1970s.^79^, ^80^ The core machinery comprises the METTL3–METTL14 writer complex,^81^, ^82^ the demethylases FTO and ALKBH5,^83^, ^84^ and YTH‐domain “reader” proteins.^85^, ^86^

**FIGURE 1:**
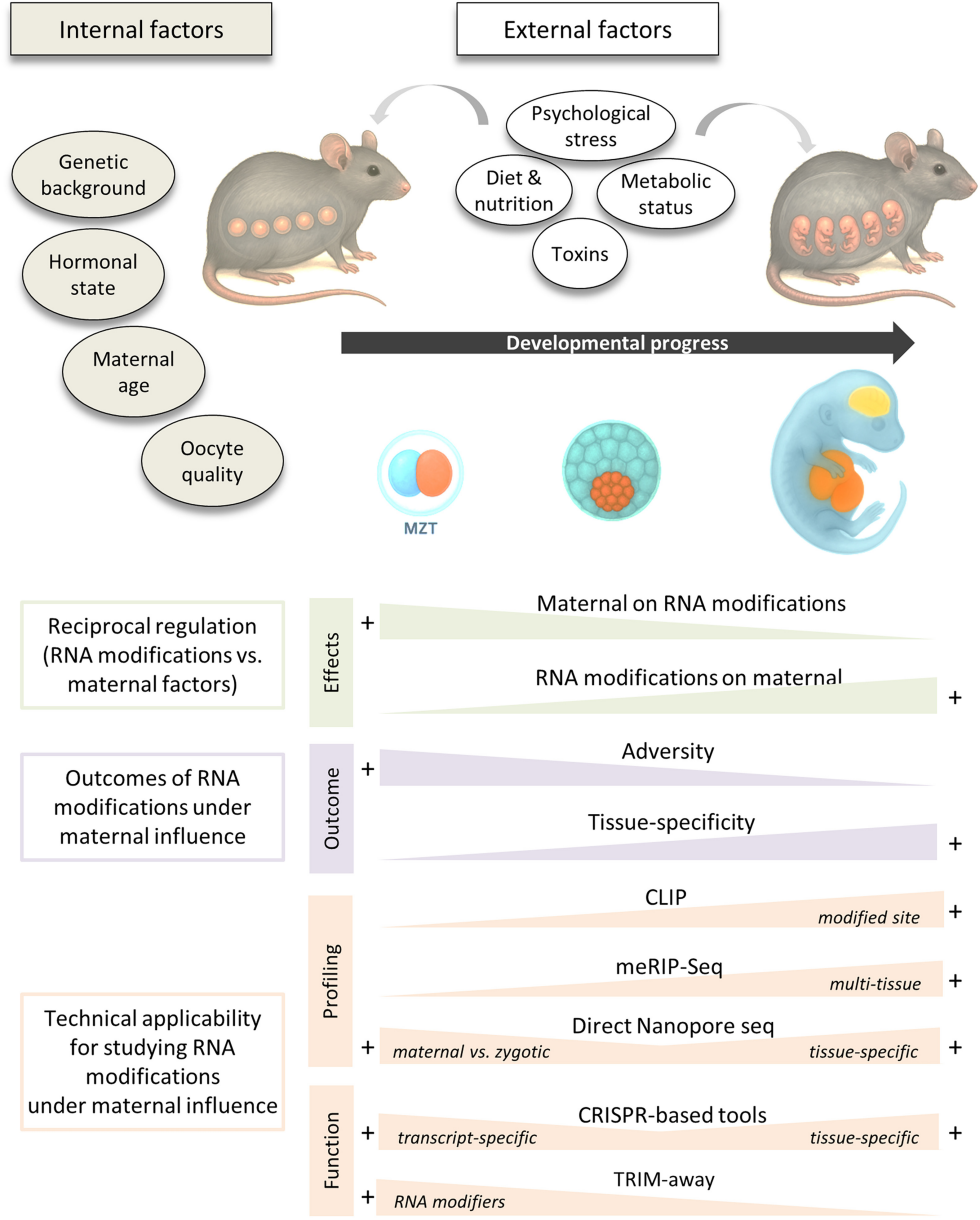
A general overview of intersections between maternal effects and RNA modifications at the developmental level.

Epitranscriptomic control is now known to be tightly integrated with developmental processes, with maternal exposure routes differing across taxa; pre‐fertilization oocyte provisioning in *ex ovo* systems versus continuous in utero exposure (oviduct, uterus, and placenta) in mammals. For example, m^6^A modifications have been shown to guide cell fate transitions during early embryogenesis in mouse embryos/ESCs, neural differentiation in mammalian systems, and skeletogenesis in zebrafish/mouse models.^74^, ^87^, ^88^, ^89^, ^90^, ^91^ By selectively destabilizing certain transcripts while stabilizing others, RNA modifications serve as molecular timers that fine‐tune gene expression in a context‐ and stage‐specific manner. These regulatory features are especially relevant in systems undergoing rapid and tightly coordinated transcriptional changes, such as the early embryo. Importantly, the deposition and removal of RNA modifications are influenced by various cellular and environmental signals.^92^, ^93^ Stress conditions, nutrient availability, heat, hypoxia, and inflammatory cues have all been shown to alter the activity or expression of RNA‐modifying enzymes.^93^, ^94^, ^95^, ^96^ This raises the possibility that external factors, such as those originating from the maternal environment, could influence the transcriptomic output of developing tissues through epitranscriptomic means.^68^, ^69^, ^70^ In this way, epitranscriptomics not only contributes to intrinsic gene regulatory logic but also offers a potential interface between environmental exposure and cellular response. Although the field is still in its early stages, advances in techniques such as MeRIP‐seq, miCLIP, single‐cell m^6^A profiling, direct RNA nanopore sequencing and live‐cell RNA biosensors can accelerate the identification of functional RNA modifications across developmental contexts.^97^, ^98^, ^99^ Future applications of these tools in maternal‐fetal models may reveal novel regulatory pathways whereby maternal signals modulate the fetal transcriptome via direct chemical modification of RNA.^100^, ^101^, ^102^ In this framework, epitranscriptomics emerges not just as a standalone regulatory system but as a candidate mediator of environmentally responsive, non‐genetic inheritance (Figure 1).

## 3 EARLY DEVELOPMENTAL WINDOWS AND MATERNAL REGULATION OF THE FETAL EPITRANSCRIPTOME

Early embryonic development is marked by a series of tightly regulated transitions, during which the embryo is highly sensitive to environmental cues, including those derived from the maternal organism. In *ex ovo* embryos these cues are primarily encoded in the oocyte/egg and perivitelline environment, whereas in mammals additional cues arise during oviductal transit, uterine residence, and placentation.^103^, ^104^ These windows of developmental plasticity offer unique opportunities for maternal signals to influence gene expression and cell fate decisions.^105^ It is being proposed that such influence may extend to the epitranscriptomic layer, wherein RNA modifications could mediate adaptive responses to maternal inputs during critical periods of embryogenesis^68^, ^90^, ^102^, ^106^ (representative examples across taxa are summarized in Table 1).

**TABLE 1:** Maternal cues, epitranscriptomic mechanisms, and outcomes across models.

Maternal cue/context	Route	Model	Window and tissue	Modification/enzyme	Mechanistic effect/target process	Outcome/phenotype	References
Maternal deposition of modified RNAs (baseline control of MZT)	Ex ovo	Zebrafish	MZT; early embryo	m^6^A–YTHDF2	Maternal mRNA clearance; timing of ZGA	Timely MZT; normal early development	58
Maternal deposition of modified RNAs (granule‐mediated decay)	Ex ovo	Fruit fly	Early embryo	m^6^A → FMR1 granule phase switch	Maternal RNA decay via RNP phase transition	Proper early developmental progression	57
Maternal deposition of modified RNAs (cytosine methylation)	Ex ovo	Fruit fly	Early embryo	m^5^C (NSUN2/NSUN6)	Cell‐cycle control and MZT timing via maternal mRNA m^5^C	Proper cleavage timing/MZT	52, 102
Oocyte maturation and early embryo control	Ex ovo	Xenopus	Oocyte maturation; early embryo	m^6^A	Translational control of maternal mRNAs	Competent oocyte maturation/early development	107
Maternal METTL3 requirement for oocyte/embryo	Direct fetal tissue	Mouse	Oocyte maturation; preimplantation	m^6^A (METTL3)	Writer activity needed for oocyte maturation and MZT	Defects in maturation/MZT when impaired	108
Maternal mettl3 function in gamete maturation	Ex ovo	Zebrafish	Gametogenesis	m^6^A (Mettl3)	Writer activity supports gamete maturation/fertility	Reduced fertility when mutated	109
Progesterone signaling at implantation	Placenta‐mediated (uterine/implantation axis)	Mouse/Human	Peri‐implantation; endometrium/trophoblast	m^6^A (METTL3; readers)	m^6^A‐mediated translation of PR and implantation factors	Implantation failure when METTL3 is deficient	110
Trophoblast invasion defects in preeclampsia	Placenta	Human	First trimester placenta; trophoblast	m^6^A (WTAP–HMGN3 axis)	m^6^A program limits invasion	Early‐onset preeclampsia association	111
Placental programs affecting FGR	Placenta	Human	Placenta; trophoblast	m^6^A (METTL3; IGF2BP2 → FOSL1)	m^6^A –reader stabilization of FOSL1	Reduced invasion; fetal growth restriction	112
Maternal obesity	Placenta	Human	Term placenta	Global m^6^A decrease (writers/erasers)	Altered placental RNA methylation landscape	Adverse fetal growth association	113
Preeclampsia case–control methylome	Placenta	Human	Placenta	m^6^A mapping differences	Pathway changes in diseased placenta	Disease‐linked m^6^A signatures	114, 115, 116
Maternal microbiome	Direct fetal tissue	Mouse	Fetal brain and intestine	m^6^A patterns	Maternal microbial metabolites modulate fetal m^6^A	Region‐specific fetal m^6^A changes	117
Maternal low‐protein diet	Direct fetal tissue	Rat	Fetal hypothalamus	m^6^A pathway genes	Altered metabolic gene regulation	Programming of metabolic pathways	68
Maternal high‐fat diet	Direct fetal tissue	Mouse	Offspring adipose and skeletal muscle (postnatal)	m^6^A dynamics	Modification changes in metabolic tissues	Long‐term metabolic effects	118
Gestational diabetes model	Direct fetal tissue	Mouse	Offspring liver	m^6^A (RBM15 → CLDN4)	m^6^A‐mediated regulation of tight junction gene	Reduced hepatic insulin sensitivity in offspring	119
Maternal heat stress	Direct fetal tissue	Pig	Neonatal adipose	m6A changes	m6A‐linked modulation of fat deposition	Altered early fat deposition	120
High temperature during early embryogenesis	Direct fetal tissue	Pig	Early embryo	m^6^A; FTO dynamics	Temperature‐driven m^6^A changes	Reduced embryonic competence	121
Gestational arsenic exposure	Placenta → fetal	Mouse	Placenta → fetus	m^6^A (Cyr61)	Placental m^6^A on implantation/growth regulator	Placental and fetal developmental effects	122
Maternal EV‐mediated signaling	Placenta‐mediated/direct delivery	Bovine/human (in vitro)	Endometrium ↔ blastocyst; early embryo	EV cargo (RNAs, RBPs)	Transfer of RNA/RBP to conceptus	Modulation of implantation/early development	123, 124, 125
Transgenerational effect of maternal hypertension	Mixed (placenta + fetal)	Rodent	Development → adult offspring	m^6^A changes	Altered vascular injury response via m^6^A	Aggravated vascular dysfunction in adult males	126

In externally developing species, maternal effects are primarily encoded via oocyte provisioning of RNAs and their epitranscriptomic marks (m^6^A/m^5^C) and by egg‐associated environments; mechanistic studies demonstrate m^6^A–YTHDF2‐dependent maternal mRNA clearance in zebrafish and m^6^A‐instructed FMR1 granule phase switching in *Drosophila*.^57^, ^58^ Functional redundancy among zebrafish YTHDF readers also buffers aspects of maternal m^6^A control.^127^ In mammals, additional maternal inputs occur after fertilization via oviductal and uterine secretions and the placenta, extending opportunities to modulate RNA‐modifying enzymes and fetal epitranscriptomic states. Several lines of evidence support the idea that maternal environments can shape the molecular landscape of the early embryo in a lasting manner.^128^, ^129^, ^130^, ^131^ Maternal diet, metabolic state, microbiome, and environmental stressors and chemicals modulate the expression and activity of genes and enzymes involved in RNA metabolism and modification. In mammals, two complementary routes are distinguished: (i) a placenta‐mediated axis, in which trophoblast/placental epitranscriptomics are altered and fetal supply and signaling are secondarily affected, and (ii) a direct fetal tissue axis, in which maternal cues modify epitranscriptomic machinery within fetal organs, with effects on embryonic and placental development.^68^, ^113^, ^117^, ^122^, ^126^, ^132^, ^133^, ^134^, ^135^, ^136^, ^137^, ^138^, ^139^, ^140^ For instance, maternal protein restriction in rat has been associated with altered m^6^A methylation in the developing fetal hypothalamus, coinciding with changes in metabolic gene expression.^68^ On the other hand, maternal obesity has been shown to dramatically affect m^6^A mRNA profile in human placenta with adverse effects on fetal growth.^113^ Another study in human showed that maternal stress and psychological disturbances can lead to substantial changes in fetal m^6^A RNA modification patterns in association with developmental growth.^137^ These findings suggest that maternal nutritional status may modulate the fetal epitranscriptome, potentially leading to persistent physiological consequences (Box 1).

BOX 1 **Key concepts referenced in this article.**

**Maternal effects:** Influences of a mother's genotype (genetic maternal effects) and/or phenotype/environment (non‐genetic maternal effects) on offspring phenotype; may act via maternally supplied gene products and maternal‐state–dependent cues.

**Genetic maternal effects:** Offspring phenotype determined by the maternal genotype through maternally expressed transcripts/proteins deposited during oogenesis and early embryogenesis.

**Non‐genetic maternal effects:** Influences arising from the maternal environment/physiology/behavior (e.g., hormones, metabolites, cytokines, extracellular vesicles) during oogenesis, gestation, or early postnatal life can modulate RNA‐modifying enzymes and RNA marks in the conceptus.

**Ex ovo vs in utero maternal influence:** *Ex ovo*—maternal inputs are primarily pre‐packaged in the oocyte/egg and egg‐associated environments; in utero—additional post‐fertilization exposures occur via the oviduct, uterine milieu, and placenta.

**Placenta‐mediated vs direct fetal tissue influence:** Placenta‐mediated—maternal cues reprogram trophoblast/placental epitranscriptomic machinery, secondarily altering fetal supply/signals; direct fetal tissue—maternal cues traverse the placenta and act within fetal organs to modify local RNA‐modification pathways.

**Epitranscriptomics:** Study of chemical modifications on RNA that regulate its function post‐transcription.

**m** ^**6**^ **A (N6‐methyladenosine):** The most abundant mRNA modification; affects RNA stability, splicing, and translation.

**RNA‐modifying enzymes:** Proteins that install (writers), remove (erasers), or recognize (readers) RNA modifications.

**Maternal RNAs:** Transcripts deposited into the oocyte, guiding early embryonic development.

**Maternal‐to‐zygotic transition (MZT):** Developmental phase where embryonic control shifts from maternal RNAs to the zygotic genome.

**Placental interface:** The maternal–fetal exchange site transmits nutrients, signals, and possibly RNA regulators.

**Developmental plasticity:** The capacity of an organism to alter its developmental trajectory in response to environmental cues.

**Non‐genetic inheritance:** Transmission of traits across generations independent of DNA sequence changes.

**Single‐cell epitranscriptomics:** Techniques that profile RNA modifications at the single‐cell level for spatial and temporal precision.

**TRIM‐away:** A proteolysis method used to rapidly degrade endogenous proteins, including RNA‐modifying enzymes.

**Phenotypic programming:** The process by which environmental factors during development shape long‐term traits.

Timing appears to be a key factor in this interaction. During pre‐implantation development, the zygote undergoes maternal‐to‐zygotic transition (MZT), during which maternal RNAs are degraded and zygotic transcription begins; major ZGA occurs at the two‐cell stage in mouse and around the eight‐cell stage in human. In zebrafish, by contrast, MZT is coupled to the midblastula transition (MBT), which occurs at approximately the 10th cleavage cycle (∼1000‐cell stage), when bulk ZGA is initiated, shaping when maternal epitranscriptomic influences can act in ex ovo versus in utero contexts.^58^, ^141^ It is during this phase that maternal signals, including metabolites and hormones, could influence the expression or activity of RNA‐modifying enzymes, as shown for METTL3‐dependent m^6^A in mouse oocytes/embryos, and for cytoplasmic polyadenylation control of maternal transcripts in zebrafish.^62^, ^67^, ^108^, ^142^, ^143^, ^144^ In later stages, such as gastrulation and organogenesis,^90^, ^145^, ^146^ tissue‐specific m^6^A RNA modification patterns may also be shaped by maternal conditions, with direct fetal tissue modulation observed in mouse liver (postnatal; sphingolipid/m^6^A programs), rat fetal hypothalamus (protein restriction), and mouse fetal brain/intestine (maternal microbiome), alongside distinct placenta‐mediated effects in placental compartments.^118^, ^119^, ^120^, ^147^, ^148^, ^149^, ^150^ Furthermore, the placenta itself represents a dynamic interface between maternal and fetal environments.^151^ Epitranscriptomic regulation (m^6^A) in human and mouse placenta has been increasingly recognized, including trophoblast cell studies and human trophoblast stem cells showing METTL3‐dependent programs, and patient placentas from preeclampsia cohorts, with studies identifying m^6^A‐dependent pathways that affect nutrient transport, vascular development, and immune modulation.^102^, ^111^, ^114^, ^115^, ^116^, ^149^, ^152^, ^153^ For clarity, placenta‐mediated influence refers to changes in trophoblast/placental epitranscriptomic machinery (e.g., METTL3–METTL14, FTO/ALKBH5, YTH readers) that secondarily reprogram fetal physiology, whereas direct fetal tissue influence refers to maternal cues acting within fetal organs (e.g., brain, liver, hypothalamus) to modify local RNA‐modifying enzymes and targets. Given the placenta's role in interpreting and relaying maternal cues, it is plausible that epitranscriptomic changes in placental RNA may also reflect or mediate maternal effects. Collectively, these findings support a model in which maternal influences on the fetal epitranscriptome are not only possible but likely occur during defined developmental windows when cells are transcriptionally and metabolically primed for such modulation. Investigating these windows further could yield insights into how early environmental exposures are translated into molecular phenotypes, with consequences for long‐term health and development.

## 4 POTENTIAL MEDIATORS: MATERNAL RNAs AND RNA‐MODIFYING ENZYMES

A central question in linking maternal effects to epitranscriptomic regulation is identifying the molecular mediators responsible for transmitting information from the mother to the embryonic RNA landscape. Two highly plausible routes have been proposed: maternal RNAs deposited during oogenesis and the regulation of RNA‐modifying enzymes in the embryo or placenta (mechanistic instances and routes are compiled in Table 1). Maternal transcripts orchestrate early developmental events and, in many species, persist beyond ZGA. For example, in oviparous models such as *Drosophila* and zebrafish, maternally supplied RNAs and proteins frequently perdure through gastrulation and even segmentation, whereas in mammals, where ZGA occurs earlier, most maternal transcripts are cleared sooner via regulated deadenylation and decay.^53^, ^54^, ^55^, ^56^, ^58^, ^154^ Large‐scale maternal‐effect screens in *Drosophila* and zebrafish are consistent with this view.^5^, ^6^, ^7^, ^8^, ^9^ Recent studies have suggested that these maternal RNAs are not merely inert templates but may carry post‐transcriptional modifications such as m^6^A, demonstrated in Xenopus oocytes, mouse oocytes/embryos, *Drosophila* embryos, and zebrafish during MZT, as well as maternal mRNA m^5^C in *Drosophila*, which influence their translation efficiency, subcellular localization, and degradation kinetics.^52^, ^57^, ^58^, ^66^, ^67^, ^107^ Thus, the maternal RNA pool may encode regulatory information both in sequence and in epitranscriptomic form, with reader‐mediated control exhibiting species‐specific features; for example, zebrafish YTHDF reader redundancy.^127^

**FIGURE 2:**
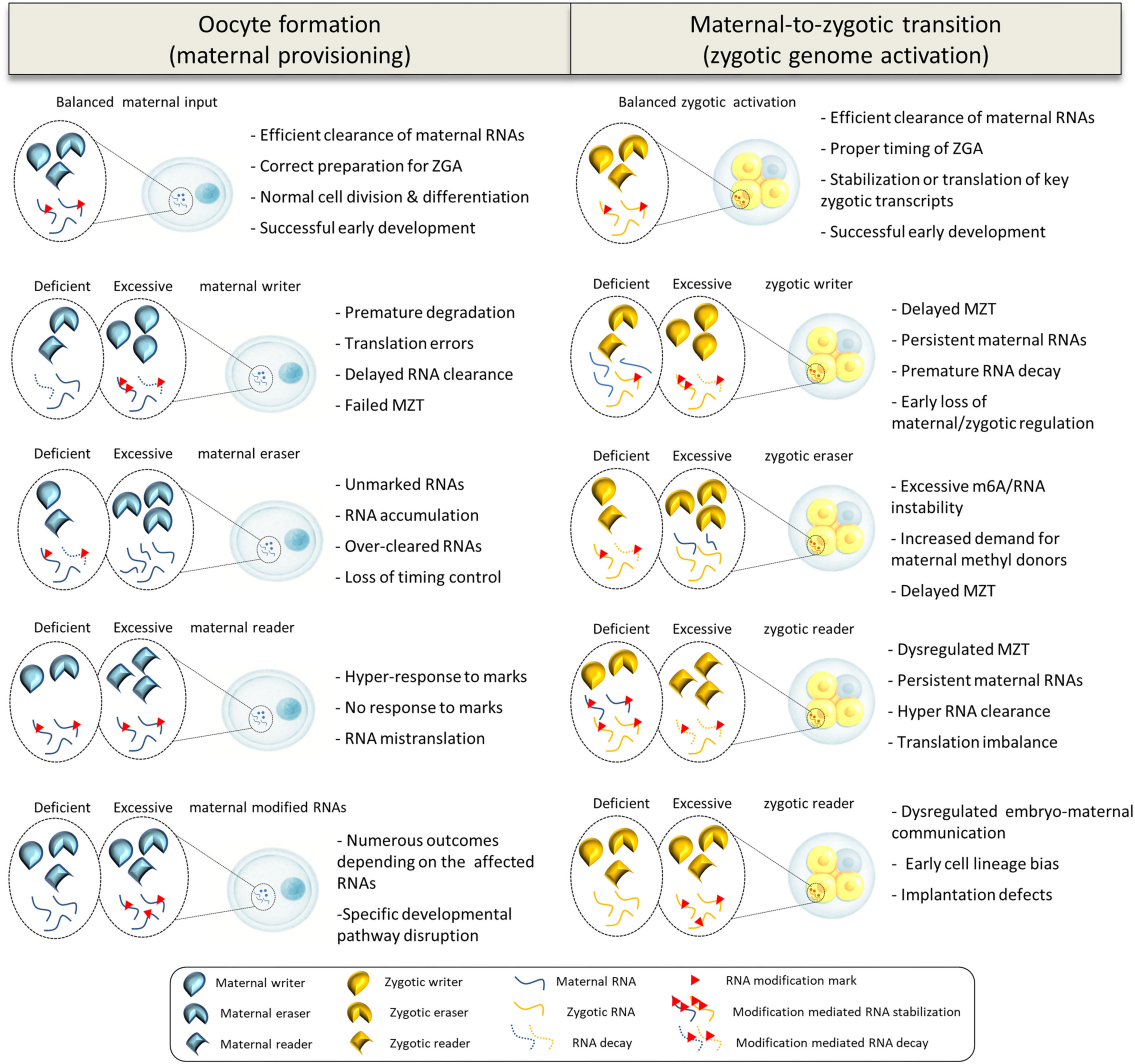
Potential outcomes for regulatory influence of maternal versus zygotic RNA modification machinery in early developmental stages.

In parallel, maternal influences on the enzymatic machinery responsible for RNA modifications have also been noted. For example, the expression of METTL3, a major m^6^A writer enzyme, has been implicated in both routes: placenta‐mediated regulation during implantation/trophoblast programs in mouse/human systems and direct fetal tissue regulation during murine oocyte/embryo MZT and organogenesis.^57^, ^108^, ^109^, ^110^, ^126^ Similarly, maternal stress has been linked to altered expression of FTO, an m^6^A demethylase, in fetal tissues such as porcine embryos under heat stress and human chorionic villi at the maternal–fetal interface.^100^, ^121^ Maternal factors can influence the abundance or activity of RNA‐modifying enzymes via three routes: (i) peri‐conception and pre‐implantation exposure to oviductal and uterine secretions; (ii) a placenta‐mediated pathway in which trophoblast/placental epitranscriptomic machinery is reprogrammed and fetal supply lines are altered; and (iii) direct fetal tissue pathways involving transplacental transfer of hormones, metabolites, cytokines, and extracellular vesicles (EVs) that act within fetal organs. The possibility that maternal hormones, cytokines, or metabolites affect the abundance or activity of these RNA‐modifying enzymes presents a promising avenue for mechanistic studies (see Figure 2 for different potential scenarios). Moreover, recent work has shown that EVs released from maternal tissues can carry RNAs and RNA‐binding proteins into fetal circulation, for example, bovine blastocyst/endometrium co‐culture and human blastocyst uptake of endometrial EVs.^123^, ^124^, ^125^ These findings support both a placenta‐mediated exchange (maternal–endometrial–trophoblast) and direct fetal tissue exposure to epitranscriptomically active cargo. Though largely speculative at present, this mechanism would provide a direct route for maternal epitranscriptomic signals to reach developing fetal tissues. Taken together, these mechanisms underscore the plausibility that maternal effects could influence offspring development via modulation of RNA modifications, either through the deposition of modified RNAs or the regulation of RNA‐modifying enzymes. As tools for detecting and manipulating RNA modifications become more precise, these hypotheses are increasingly testable and could reveal a new class of maternally derived regulatory cues in development.

## 5 EVIDENCE FOR MATERNAL‐EPITRANSCRIPTOMIC CROSSTALK: WHAT DO WE KNOW SO FAR?

While the direct regulatory connections between maternal effects and epitranscriptomics are still emerging, several independent lines of research support the plausibility of their interaction (Table 1). Early clues come from models in which maternal conditions alter RNA methylation landscapes in embryonic and fetal tissues. In one notable example, the maternal microbiome was shown to affect m^6^A patterns in the developing mouse fetal brain and intestine of mouse fetuses, illustrating a direct fetal tissue route in which maternal metabolites/signals reach and reprogram fetal epitranscriptomic machinery.^117^ This finding provided one of the first functional demonstrations of maternal influence on the fetal epitranscriptome. Further supporting evidence arises from maternal nutritional studies.^18^, ^68^, ^117^, ^155^, ^156^ For instance, maternal methionine supplementation, known to affect methyl group availability, has been associated with shifts in fetal brain m^6^A levels; this implies that methyl donors in the maternal diet could influence the activity of methyltransferase complexes.^156^ Similarly, maternal exposure to a low‐protein diet in rats (fetal hypothalamus) and a high‐fat diet in mice (offspring adipose/skeletal muscle and liver) has been linked to altered expression of m^6^A writers and erasers in both placental and fetal tissues, with downstream consequences for metabolic gene expression and differentiation outcomes.^18^, ^68^, ^117^, ^155^, ^157^

Stress and inflammation in the maternal organism have also been associated with epitranscriptomic changes in offspring.^69^, ^102^, ^130^, ^140^, ^158^, ^159^ Inflammatory cytokines, glucocorticoids, and oxidative stress, factors commonly elevated during gestational stress, can influence the abundance of m^6^A RNA‐modifying enzymes and alter the translation of key regulatory transcripts in human placenta.^112^, ^160^ These stress‐responsive changes have been observed in brain, liver, and hematopoietic systems during fetal development in mice, and in human placenta^140^, ^160^; they point toward an environmentally sensitive epitranscriptomic layer that may reflect maternal physiological states. Although most of these studies are correlational, they set the stage for more mechanistic investigations. A major limitation so far has been the difficulty in temporally and spatially resolving RNA modifications in early development. However, innovations in RNA immunoprecipitation and sequencing, combined with targeted gene editing tools, are beginning to overcome these barriers.^161^, ^162^ Single‐cell m^6^A mapping, for example, has allowed researchers to track RNA methylation states across cell types and developmental stages in vivo in mouse tissues^163^, ^164^; while still technically challenging, it holds promise for tracing maternal signal‐dependent changes in RNA modification patterns. The accumulating evidence underscores a growing consensus that maternal conditions do influence the epitranscriptomic state of offspring; however, the mechanistic links remain to be fully clarified. The current data justify targeted experiments to determine whether these RNA modifications are merely correlative signatures of maternal states or functionally involved in transmitting phenotypic effects across developmental time.

## 6 EVOLUTIONARY AND ECOLOGICAL IMPLICATIONS OF MATERNAL EPITRANSCRIPTOMIC INFLUENCE

If maternal regulation of offspring epitranscriptomics proves to be a functional mechanism, it could represent a powerful form of developmental plasticity,^89^, ^165^ enabling rapid phenotypic tuning in response to environmental variability without requiring genetic change.^93^, ^94^, ^166^ From an evolutionary standpoint, such a system would provide a mechanism for the transgenerational transmission of adaptive traits in response to maternal environmental cues,^144^, ^167^ particularly under conditions where environmental conditions fluctuate more rapidly than genetic adaptation can occur.^93^ In ecological systems, maternal effects have often been interpreted as anticipatory mechanisms, preparing offspring for expected environmental conditions based on maternal experience.^14^, ^15^ Epitranscriptomic modifications, being reversible yet stable across short developmental windows, may offer an ideal molecular substrate for such transient, tunable responses.^165^, ^168^ By modulating RNA stability and translation in specific tissues, maternal environments might prime offspring physiology or behavior in context‐specific ways: for example, enhancing stress resilience, altering metabolic capacity, or modifying developmental timing.^102^, ^169^, ^170^, ^171^ This potential utility is further amplified in species with external development, where maternal investment through the oocyte becomes the primary avenue for environmental signaling.^172^, ^173^, ^174^ In these systems, maternal provisioning of modified RNAs or regulators of RNA modification may serve as early warning systems, tuning gene expression programs in advance of environmental exposure.

Despite these possibilities, the evolutionary dynamics of RNA modification systems remain poorly understood. Few studies have assessed the heritability, variability, or fitness consequences of altered RNA modification profiles across generations.^126^, ^139^, ^175^ If maternal epitranscriptomic regulation is indeed a widespread mechanism, it may challenge conventional models of non‐genetic inheritance by providing a rapid, semi‐stable, and reversible method for transferring information from mother to offspring. Furthermore, different species may rely on distinct epitranscriptomic strategies based on their reproductive biology, embryonic timing, or environmental unpredictability. Comparative studies across taxa, especially those with contrasting maternal provisioning modes,^176^ will be essential for understanding how and why such mechanisms may have evolved. These questions open up not only new empirical challenges but also opportunities to refine theoretical models of inheritance, adaptation, and plasticity.

## 7 EXPERIMENTAL CHALLENGES AND EMERGING TOOLS TO UNCOVER MECHANISTIC LINKS

Investigating the potential mechanistic connections between maternal effects and epitranscriptomic regulation presents significant experimental hurdles, primarily due to the dynamic and context‐specific nature of both systems. First, RNA modifications are often transient and highly cell‐type‐specific; detecting these marks in early embryos, which are composed of rapidly dividing and differentiating cells, requires both spatial and temporal resolution that few current methods fully provide. Another challenge lies in distinguishing whether observed RNA modifications are a direct consequence of maternal signals or arise as secondary responses during embryonic development. This distinction necessitates experimental systems that allow precise manipulation of maternal environments while tracking downstream molecular consequences in fetal tissues at defined stages.

To address these challenges, a growing set of molecular tools has been applied. Antibody‐based techniques such as MeRIP‐seq and miCLIP have enabled transcriptome‐wide mapping of RNA modifications, particularly m^6^A.^98^ These methods, though powerful, are limited by antibody specificity and often require high input material, making them less suitable for early developmental samples. More recently, the development of single‐cell epitranscriptomic methods has begun to fill this gap, allowing for resolution of RNA modifications at the level of individual cells or lineages.^177^ In addition, the advent of direct RNA nanopore sequencing has enabled transcriptome‐wide profiling of m^6^A modifications without the need for chemical conversion or enrichment, offering a powerful advantage for studying RNA methylation at the zygotic or fetal stage with single‐molecule precision.^99^ In parallel, CRISPR‐based tools have been adapted to target RNA methylation enzymes to specific transcripts; although still in early development, these systems allow direct testing of the functional role of epitranscriptomic marks in gene regulation and development.^178^, ^179^ Of particular relevance to maternal–fetal studies is the application of TRIM‐away, a proteolysis‐based technique that enables acute depletion of specific endogenous proteins, including RNA‐modifying enzymes.^180^ TRIM‐away has proven effective in depleting RNA methyltransferases or demethylases in zebrafish and Xenopus embryos without genetic manipulation, offering a rapid and reversible method for testing the requirement of these enzymes during sensitive developmental windows.^180^, ^181^, ^182^ This tool may be especially valuable for dissecting the temporal dynamics of maternal influences on the fetal epitranscriptome. Despite these advancements, technical and interpretive challenges remain. Cross‐reactivity of antibodies, the functional redundancy of RNA modification enzymes, and the incomplete understanding of modification “readers” complicate the attribution of phenotypic effects to specific molecular events. Nevertheless, with the continuing refinement of tools and model systems, the field is well‐positioned to explore how maternal cues may operate through RNA modifications to shape developmental trajectories.

## 8 CONCLUDING THOUGHTS: CONCEPTUAL BRIDGES AND OPEN QUESTIONS

The possibility that maternal effects and epitranscriptomic regulation intersect to shape offspring development presents a compelling framework for rethinking non‐genetic inheritance. While both domains have been extensively studied in isolation, their potential integration opens a new dimension in developmental biology, one where environmental cues from the maternal organism could be translated into post‐transcriptional regulatory changes in the offspring, with implications for phenotype, health, and even evolutionary fitness. This perspective article has outlined several lines of evidence that support such a connection; however, it must be emphasized that definitive mechanistic links remain largely speculative at present. Observations that maternal nutritional, microbial, or stress‐related environments alter RNA modification patterns in fetal tissues are intriguing, yet causality has not been consistently demonstrated. Whether these RNA modifications are central mediators or merely responsive markers remains unresolved in many contexts.

Key questions remain to be addressed. For instance, how specific and selective are maternal influences on RNA modification machinery? Are these changes preserved through cell divisions, and can they be transmitted beyond one generation? Which transcripts are most susceptible to maternal modulation, and how do these modifications intersect with other regulatory layers such as microRNAs, alternative splicing, or chromatin state? Furthermore, to what extent do these interactions vary across tissues, developmental windows, or species?

Addressing these questions requires multidisciplinary collaboration; developmental biologists, molecular geneticists, and RNA biochemists must work in concert with systems biologists and evolutionary theorists to establish a common language and set of priorities. The development of temporally controlled, tissue‐specific tools, such as TRIM‐away, inducible CRISPR interference systems, and advanced imaging of modified RNAs in vivo, will be essential to disentangle maternal inputs from autonomous embryonic processes. Moreover, more comparative studies across model organisms and ecological systems are needed to test the generality, adaptability, and interactomics of this regulatory axis.^183^ The purpose of this article has not been to assert a definitive model but rather to highlight an underexplored conceptual space that may prove fruitful for future research. By framing maternal effects and epitranscriptomics as potentially interconnected systems, the goal is to stimulate discussion, generate testable hypotheses, and encourage researchers in both fields to consider overlapping mechanisms. Whether or not maternal regulation of the offspring epitranscriptome proves to be widespread or only context‐specific, the exploration of this intersection promises to deepen our understanding of how environment, regulation, and inheritance are woven together in shaping organismal development. In conclusion, the maternal–epitranscriptomic axis, while still in its theoretical infancy, offers a promising conceptual bridge in developmental biology—one that may ultimately transform how we understand phenotypic plasticity, intergenerational signaling, and the molecular logic of early life.

## FUNDING INFORMATION

The author received no specific funding for this work.

## CONFLICT OF INTEREST STATEMENT

The author declares that he has no competing interests.

## Data Availability

Data sharing not applicable to this article as no datasets were generated or analysed during the current study.
